# Observing hidden neuronal states in experiments

**DOI:** 10.1371/journal.pcbi.1013748

**Published:** 2025-12-08

**Authors:** Dmitry Amakhin, Anton Chizhov, Guillaume Girier, Mathieu Desroches, Jan Sieber, Serafim Rodrigues

**Affiliations:** 1 Laboratory of Molecular Mechanisms of Neural Interactions, Sechenov Institute of Evolutionary Physiology and Biochemistry of RAS, Saint Petersburg, Russia; 2 Institute for Theoretical Physics, University of Bremen, Bremen, Germany; 3 MCEN Team, Basque Center for Applied Mathematics, Bilbao, Spain; 4 MathNeuro Team, Inria Branch of the University of Montpellier, Montpellier, France; 5 College of Engineering, Mathematics and Physical Sciences, University of Exeter, Exeter, United Kingdom; 6 Ikerbasque, The Basque Science Foundation, Bilbao, Spain; University of Edinburgh, UNITED KINGDOM OF GREAT BRITAIN AND NORTHERN IRELAND

## Abstract

In this article we demonstrate a general protocol for constructing systematically experimental steady-state bifurcation diagrams for electrophysiologically active cells. We perform our experiments on entorhinal cortex neurons, both excitatory (pyramidal neurons) and inhibitiory (interneurons). A slowly ramped voltage-clamp electrophysiology protocol serves as closed-loop feedback controlled experiment for the subsequent current-clamp open-loop protocol on the same cell. In this way, the voltage-clamped experiment determines dynamically stable and unstable (hidden) steady states of the current-clamp experiment. The transitions between observable steady states and observable spiking states in the current-clamp experiment provide partial evidence for stability and bifurcations of the steady states. This technique for completing steady-state bifurcation diagrams in a model-independent way expands support for model validation to otherwise inaccessible regions of the phase space. Overlaying the voltage-clamp and current-clamp protocols leads to an experimental validation of the classical *slow-fast dissection* method introduced by J. Rinzel in the 1980s and routinely applied ever since in order to analyse slow-fast neuronal models. Our approach opens doors to observing further complex hidden states with more advanced control strategies, allowing to control real cells beyond pharmacological manipulations.

## Author summary

We demonstrate a general protocol for constructing systematically experimental steady-state bifurcation diagrams of neurons. We test it with both excitatory (pyramidal neurons) and inhibitory (interneurons) cells from the entorhinal cortex of rats. A slowly ramped voltage-clamp electrophysiology protocol serves as closed-loop feedback controlled experiment for the subsequent current-clamp open-loop protocol on the same cell. In this way, the voltage-clamped experiment determines dynamically stable and unstable (hidden) steady states of the current-clamp experiment. The transitions between observable steady states and observable spiking states in the current-clamp experiment provide partial evidence for stability and bifurcations of the steady states. This technique for completing steady-state bifurcation diagrams in a model-independent way expands support for model validation to otherwise inaccessible regions of the phase space. We explain why the method works by using multiple-timescale dynamics arguments.

## Introduction

When characterising the dynamics of nonlinear systems, one fundamental criterion for a model is if its stable states such as stationary solutions or periodic orbits match experimental observations. The ability to fit and validate models is, thus, greatly expanded by experimental tools with the capacity to unveil non-observable (sensitive or dynamically unstable) states that are otherwise inaccessible to standard measurements. The combination of observable and non-observable states gives access to an experimental equivalent of parameter-dependent families of stable and unstable states in a model, which are usually referred to as a bifurcation diagram.

This article applies a new experimental technique of using feedback control to find unstable states to electrophysiology experiments on neuronal cells. Our aim is to support systematic validation of neuron models by comparing bifurcation diagrams and observing their between-cell variability. We focus on unstable parts of input-dependent families of steady-state solutions obtained by feedback-controlled experiments and compare them with indirect evidence from standard measurements from open-loop experiments. This extends recent work of Ori et al. [1,2] constructing phase diagrams from neuronal data, and complements other approaches such as using data to verify the bifurcation structure of neuronal models [3,4], model-based data analysis [5] or parameter estimation from data [6].

Our technique is a simplification of the so-called *control-based continuation (CBC)* method, an approach which has been recently demonstrated in mechanical experiments [7], vibrations and buckling experiments [8], pedestrian flow experiments [9], atomic-force microscopy [10], cylindrical pipe flow simulations [11], and feasibility studies for synthetic gene networks [12,13]. Indeed, CBC is a procedure that combines feedback control [14] and pseudo-arclength continuation [15] in a model-free environment, that is, only reliant upon noisy experimental data in a closed-loop control setup. The objective is to compute experimental bifurcation diagrams, that is, both families of stable and unstable states (either stationary or periodic) together with bifurcation points joining such families. Importantly, the control is noninvasive in the sense that it vanishes once an equilibrium or periodic of the uncontrolled system has been reached; the control is decreased iteratively using a Newton’s method. In our case, the approach is simpler and does not require the use of Newton iterations since we consider a slow ramp on the control target. Hence we exploit de presence of two timescales in the experimental procedure in order to obtain directly an approximation of the steady-state bifurcation curve, and we explain why such a bifurcation curve is obtained at low cost; see below. Our approach is hence useful for both modelers and experimentalists, as it relies upon standard protocols routinely used in patch-clamp electrophysiology. It can further decipher the excitability class of a given real neuron as well as help fitting a model to data by using both current-clamp and voltage-clamp protocols.

**Fig 1:**
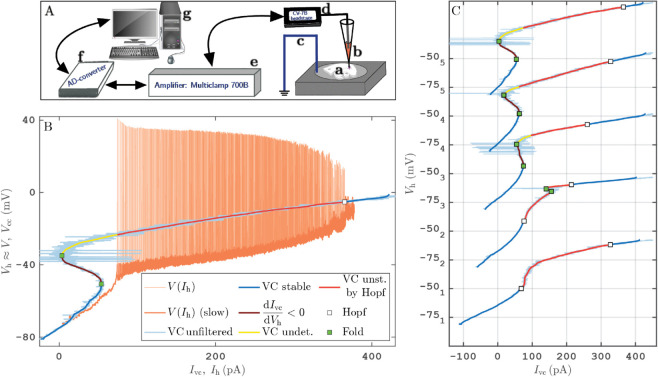
A: Sketch of experimental setup with brain slice (**a**), patch pipette (**b**), reference electrode (Ag-AgCl pellet) connected to ground (**c**), amplifier (**e**, *Multiclamp 700B*) with CV-7B headstage (**d**), AD-converter (**f**, *National Instruments NI USB-6343*) and standard PC computer (**g**, https://pixabay.com/vectors/computer-desktop-workstation-office-158675/). B: VC and CC protocol runs for cell 5: $\left(I_{vc}(t),V_{h}(t)\right)$-curve for VC run (thin bright blue: unfiltered data with sampling time step $5\times 10^{-5}$ s, blue/red/brown: median of $I_{vc}$ over moving windows of size $\Delta w=4\times 10^{3}$ steps equalling 0.2s) and $\left(I_{h}(t),V_{cc}(t)\right)$-curve for CC run (orange, mean of $I_{h}$ over moving windows. C: $\left(I_{vc}(t),V_{h}(t)\right)$-curves for VC protocol of all 5 cells on waterfall $V_{h}$-axis (color coding indicates conjectured stability as indicated for (b)).

In our electrophysiology experiments on entorhinal cortex neurons we treat the cell as an electric circuit, and apply a voltage clamp (VC) [16,17], followed by current clamp (CC). In the VC setup the electrode acts as a voltage source at the neural membrane, fixing the potential across the neural membrane, measuring the current, while the CC setup adds a fixed external current, measuring the resulting membrane potential; see the illustration for the experimental setup in Fig 1A and the S1 Text for further details. In-vivo neurons are subject to current signals that drive spiking (oscillatory) or rest (steady) states of the neural membrane potential [18]. This makes the CC setup the open-loop part of the protocol. In contrast, the VC part of our experiment is the closed-loop feedback-controlled part of the protocol because the voltage source regulates the external current to maintain the hold voltage. VC has been applied successfully to study neuronal nonlinear current-voltage relationships, so-called *N-shaped I-V characteristics*, which cause enhanced neuronal excitability and influence the regenerative activation of certain ionic currents (e.g., sodium) [19–23] across the neural membrane, into and out of the cell. We show that the VC protocol with a slowly varied reference voltage signal gives access to stable and unstable neuronal steady states of the neuron, which was hinted at in [24,25]. In contrast, the open loop CC protocol with a slowly varying applied current always follows stable (observable) states, driving the neuron to dynamically transition between its observable rest states and its observable spiking states.

We interpret these combined experimental protocols (VC and CC) using multiple-timescale dynamics, in particular, the dissection method [26], which reveals the dynamic bifurcations in the experiment, as demonstrated in the experimental bifurcation diagram in Fig 1B. In this slow-fast framework, the states traced by the VC protocol with slow variation correspond to the steady-state experimental bifurcation diagram of the so-called *fast subsystem* of the mathematical model describing the protocol [26], that is, the model system with constant input current. Hence, the N-shaped I-V relation of a neuron should be seen as a S-shaped V-I bifurcation diagram, such as in Fig 1B.

Following this strategy, we demonstrate the feasibility of tracking a family of neuronal steady states (stable and unstable) via variations of reference signal and reparameterizing the obtained curve using the feedback current.

## Results

The experimental neuronal bifurcation diagram in Fig 1B shows the time profiles $\left(I_{h}(t),V_{cc}(t)\right)$ of the CC protocol run (orange, thin) and $\left(I_{vc}(t),V_{h}(t)\right)$ of the VC protocol run (bright blue, thin) for cell 5 overlaid in the (*I*,*V*)-plane. For both runs $I_{h}(t)$ and $V_{h}(t)$ were slowly increased, respectively (see Materials and Methods). After smoothing, the VC time profile is the S-shaped curve $\left(I_{vc,sm}(V_{h}),V_{h}\right)$ (blue/brown/red, thick). It equals the (*I*,*V*)-characteristic of the stationary neuronal states of the CC protocol, *including dynamically unstable states* (brown and parts of red). The transition to stable spiking states is compatible with (Hodgkin) class-I excitability, however we do not have sufficient data to distinguish the different classes of excitability. See Fig B in S1 Text for how the interspike intervals depend on $I_{h}$.

The dynamical stability and instability of stationary states is inferred based on two pieces of evidence: (i) the negative slope of the $\left(I_{vc,sm},V_{h}\right)$-curve (brown) after smoothing over a moving window with larger size ($\Delta w=1.5\;\times \;10^{4}$ steps equaling  = 0.75s in Fig 1C), or (ii) by the presence of slow-fast oscillations (neuronal firing) in the CC run at $I_{h}$ equalling the $I_{vc,sm}$ (red). Criterion (i) implies instability for topological reasons. Criterion (ii) provides only circumstantial evidence. In Fig 1B (cell 5) the darker shading of the (orange-colored) CC run indicates slow dynamics as identified by the norm $\left|(V_{cc}^{\prime }(t)\sqrt{\Delta_{t}},{V_{cc}^{\prime }}^{\prime }(t))\Delta_{t}\right|$ being less than a fixed threshold (after smoothing of $V_{cc}(t)$ and $V_{cc}^{\prime }(t)$, see S1 Text for detailed definition). This shading shows that the firing oscillations in Fig 1B spend most time near their voltage minimum $V_{\min}$ for the respective $I_{h}(t)$. Fig 5C and 5E show an embedding of the firing oscillations of cell 5 into the $\left(V_{cc},V_{cc}^{\prime }\right)$-plane, and a zoom near $I_{h}\approx 200$ pA. The projection and zoom indicate that the firing oscillation passes slowly near the presumed fixed point, but leaves its neighborhood again. At the low-current end of the $\left(I_{vc,sm},V_{h}\right)$-curve, the CC protocol for cell 5 shows no firing oscillations (recall that $I_{h}$ is ramped up) in Fig 1B. Thus, the part of the $\left(I_{vc,sm},V_{h}\right)$ curve between fold and presence of slow-fast oscillations is labelled “undetermined” (colored yellow) as the combination of single-ramp VC and CC protocol do not provide evidence for or against stability of this part. We do not label the “transition” from stability label “undetermined” to “unstable” as a Hopf bifurcation as the slow parts of the firing oscillations are approximately 20 mV below the equilibrium indicated by the VC run. So, it is unclear if (and where precisely) a change of stability occurs between $I_{db}\approx 365$ pA and the fold of steady states at $I_{h}\approx 3$ pA.

The stability boundaries of the stationary states are labelled as bifurcations in Fig 1B. The change of stability near the disappearance of stable spiking states at $I_{db}$ is labelled as a Hopf bifurcation. We observe that for $I_{h}>I_{db}$ small-amplitude oscillations are visible, which emerge from relaxation type oscillations with a slow phase near the stationary state for $I_{h}<I_{db}$. Fig I in S1 Text shows a zoom of Fig 1B near $I_{db}$. These features are typical for a singular Hopf bifurcation [27] as encountered also in model simulations for excitatory neurons. The fold points of the $\left(I_{vc,sm},V_{h}\right)$-curve are saddle-node (fold) bifurcations. Fig 1C shows the stationary-state curves with their inferred dynamical stability for all 5 cells (see S1 Text for CC run time profiles used to partially infer stability of cells 1–4). The cells are vertically ordered and numbered according to depth of the S-shape, determining if they fall into the category of class-I or class-II neurons. We use the same convention as in Fig 1B for parts of the curve where we have partial evidence for instability: no bifurcation is indicated at transitions between stability labels “undetermined” and “unstable”. Fig 1C demonstrates wide variability in steady-state curve shape among cells of nominally same function.

**Fig 2:**
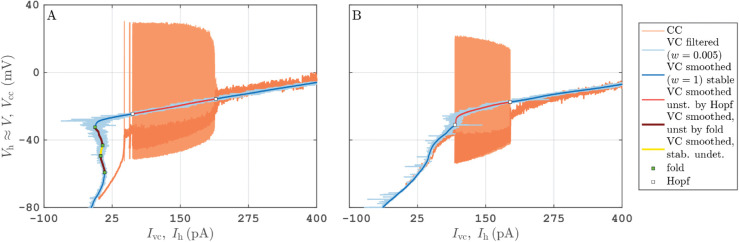
A: Experimental bifurcation diagram for an interneuron from the entorhinal cortex. B: Experimental bifurcation diagram for a class-II PY neuron from the same region. Protocols identical and color coding to Fig 1B.

**Fig 3:**
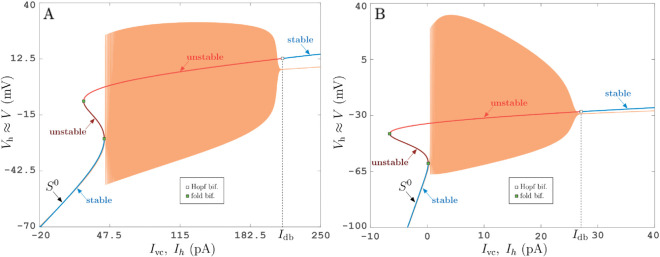
VC and CC in silico. Protocol as described for Fig 1B applied to A: a class-I Morris-Lecar neuron model (10) [33] as example of excitatory cell, and B: a class-I Wang-Buzsáki neuron model (12) [34] as example of inhibitory interneuron; see (10) and (12) for differential equations and Tables 1 and 2 for parameter values. The two-dimensional fast subsystem has a S-shaped steady-state curve satisfying steady-state conditions (4). The steady-state *I*–*V* curve (4) and the (multi-color) S-shaped curve from the VC protocol (2) are indistinguishable throughout the range of input currents $I_{vc}$. The orange curve resulting from the CC protocol is very close to *S*^0^ and the VC protocol near its dynamically stable parts. See Figs F and G in S1 Text for numerical bifurcation diagrams.

**Fig 4:**
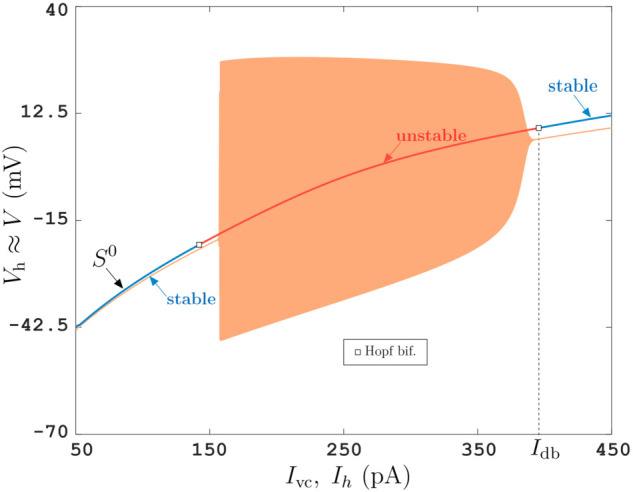
VC and CC protocols as described for Fig 1B applied to the Morris-Lecar model in a parameter regime where it behaves as a class-II neuron model. See (10) for differential equations and Table 1 for parameter values and Fig H in S1 Text for numerical bifurcation diagram.

The method described in this work is versatile and uses only standard electrophysiological procotols. Hence, it can be applied to any neuron type. The experimental bifurcation diagrams in Fig 2A illustrate this point. Panel A shows the results of the VC and CC protocols applied to an inhibitory neuron from the entorhinal cortex while panel B shows the results of same protocols applied to a class-II neuron. The steady-state curve in Fig 2A is more complicated than for PY neurons’ recordings shown in other figures. However it is compatible with a class-I excitable neuron. Since the steady-state curve in Fig 2A has multiple folds, there are some parts of the branch where we cannot determine stability safely from topology (that is, by checking that $dI_{vc,sm}/dV_{h}<0$), or from the near-by embedded trajectories of relaxation oscillations. Similar to Fig 1 we have colored the parts of the steady-state curve in Fig 2A with undetermined stability yellow, while keeping the part for which instability is suggested by existence of relaxation oscillations in red. For the range of $I_{h}$ between 43 pA and 60 pA during the CC protocol in Fig 2A we observe a stable steady state $V_{cc}$ response, with intermittent voltage spikes typical for an excitable stable steady state near a singular Hopf bifurcation subject to noise [28]. For this reason we label the low-$I_{h}$ onset of large-amplitude relaxation oscillations in Fig 2A also as a Hopf bifurcation. A potential alternative mechanism for the sudden transition of firing oscillations to steady state near $I_{db}$ (≈210 pA for Fig 2A, ≈190 pA for Fig 2B) is that the firing oscillations experience a *saddle-node of periodic orbits*. This would imply a bistability between firing and steady state for $I_{h}<I_{db}$, a scenario that has been observed near depolarization blocks in models of dopaminergic neurons by [29]. Our illustrative examples of mathematical neuron models shown in Figs 3A and 4 have such a small region of bistability (Fig 3A near $I_{db}$ and Fig 4 near $I_{h}\approx 135$ pA) for the parameters used in the computations (see Figs F, G and H in S1 Text for numerical bifurcation diagrams).

We observe that the steady-state curves for the CC run and the VC run slightly deviate from each other, which we attribute to a drift in cell properties. There are examples of scenarios with multiple folds in the steady-state curve in the literature, in particular with low-threshold spiking neurons [30].

### Analysis

To see why VC-run time profiles approximate the experimental bifurcation diagram with unstable stationary states of neurons in CC protocol, we use the formalism of multiple-timescale dynamical systems. Superimposing the data from VC and CC protocol also gives the first experimental illustration of slow-fast dissection. The effect of the respective clamps can be understood in a general class of conductance-based models for the neuron,

$$C\dot{V}=-\sum_{j}I_{j}(x_{j},V)+I_{ext},\;\tau_{j}(V){\dot{x}}_{j}=x_{\infty ,j}(V)-x_{j},$$ (1)

describing the current balance across the neuron’s membrane. The membrane potential is *V*, $I_{j}(x_{j},V{)}_{j=1,\ldots ,N}$ are the currents across different voltage-gated ion channel types and $I_{ext}$ is the external current. Each channel type *j* has a set of associated gating variables *x*_*j*_, which are possibly vectors of length *n*_*j*_ if the channel gate has both activation and inactivation states, with steady-state gating functions $x_{\infty ,j}(V)$ (also of size *n*_*j*_) and relaxation times $\tau_{j}(V)$ (a diagonal $n_{j}\times n_{j}$ matrix). The observed dynamic effects such as oscillations (firing/spiking) and negative-slope (*I*,*V*) characteristics are determined by these channel coefficients *I*_*j*_, $x_{\infty ,j}$ and $\tau_{j}$ that are traditionally obtained by parameter fitting from VC experiments, a difficult and ill-posed problem, as gating variables *x*_*j*_ are not directly measured [31].

The VC and CC protocols use different mechanisms for generating $I_{ext}(t)$. The VC protocol is a closed loop where a voltage source regulates $I_{ext}$ with high gain $g_{c}$ to achieve the slowly varying hold voltage $V_{h}$ at the voltage source for general model (1), measuring $I_{vc}$:

$$I_{ext}\approx I_{vc}=g_{c}(V_{h}-V),\;\;{\dot{V}}_{h}=\varepsilon \Delta_{V}$$ (2)

which turns general model (1) with VC protocol (2) into a multiple-timescale dynamical system with $1\;+\;\sum n_{j}$ fast state variables $\left(V,x_{j}\right)$ (where $\sum n_{j}$ is the overall number of gating variables) and one slow state variable $V_{h}$, corresponding to the feedback reference signal [32]. The speed at which $V_{h}$ varies is $\varepsilon \Delta_{V}$ with $\Delta_{V}(t)=0.183$ mV/ms, where we extract the dimensionless small factor $\varepsilon =10^{-2}$.

In contrast, the CC protocol holds $I_{ext}$, measuring the generated voltage $V_{cc}$, thus, corresponding to an open-loo system, permitting e.g. the spiking seen in Fig 1B:

$$I_{ext}\approx I_{h},\;\;V_{cc}\approx V,\;\;{\dot{I}}_{h}=\varepsilon \Delta_{I}.$$ (3)

The applied hold current $I_{h}$ is varied slowly at speed $\varepsilon \Delta_{I}$ with $\varepsilon =10^{-2}$ and $\Delta_{I}=0.66\ldots 0.75$ pA/ms. General model (1) with CC protocol (3) is also a slow-fast system with 1+N fast variables $\left(V,x_{j}\right)$ and 1 slow variable $I_{h}$. The gain $g_{c}$ in VC protocol (2) is limited by the imperfect conductance across the non-zero spatial extent of the membrane. Even though the conductance-based model (1) is for the potential *V* across the entire membrane and only $V_{h}$ at the clamp is measured, we approximate $V_{h},V_{cc}\approx V$ for the membrane potential, and $I_{h},I_{vc}\approx I_{ext}$ for the external current in (1).

Following a classical multiple-timescale approach, we consider the ε=0 limit of general model (1) with VC protocol (2), which corresponds to its $\left(1\;+\;\sum n_{j}\right)$-dimensional fast subsystem (1), with $I_{ext}=g_{c}(V_{h}\;-\;V)$, where $V_{h}$ is now treated as a parameter. For fixed $V_{h}$ and voltages in the range −80…30 mV of interest, model (1) with VC protocol (2) has only stable steady states (no limit cycles, that is, no neuronal spikes); see S1 Text for details. For a fixed hold voltage $V_{h}$, the steady states of system (1), (2) with $I_{ext}=g_{c}(V_{h}-V)$ satisfy the algebraic equations for $(V,x_{j}{)}_{j=1,\ldots ,N}$

$$\sum_{j}I_{j}(x_{j},V)=g_{c}(V_{h}-V),\;\;x_{j}=x_{\infty ,j}(V),$$ (4)

where $I_{eq}(V)=\sum_{j}I_{j}(x_{\infty ,j},V)$ is the equilibrium current for fixed membrane potential *V*. The solutions of algebraic system (4) form a (1D) steady-state curve for model (1) with VC protocol (2), which is normally hyperbolic (transversally attracting) for ε=0. For ε≠0 the increase of $V_{h}$ with speed $\varepsilon \Delta_{V}$ introduces a slow variation of all states $\left(V,x_{j}\right)$. Hence, *V* and the feedback current $I_{vc}=g_{c}(V_{h}\;-\;V)$ (as measured), are not at their steady-state values given by (4), but they are changing dynamically. This results in a difference between the measured curve $\left(I_{vc},V_{h}\right)$ in Fig 1B and the (*I*,*V*)-values of the desired steady-state *I*–*V* curve given by (4).

Geometrical singular perturbation theory (GSPT) by Fenichel [35] implies that after decay of initial transients every trajectory of the general model (1) with VC protocol (2) satisfies the algebraic conditions (4) for the steady-state curve up to order *ε*. The first-order terms in *ε* are

$$I_{eq}(V)-I_{vc}(V)\approx I_{eq}^{\prime }(V)\dot{V}\;\frac{\tau_{1/2}}{\ln2}≲I_{eq}^{\prime }(V_{h})0.2\text{ mV},$$ (5)

where $\tau_{1/2}$ is the time for deviations from the transversally stable steady-state *I*–*V* curve (4) to decay to half of their initial value; see eq (7) and Fig C in S1 Text for details. We estimate $\tau_{1/2}$ from recovery transients after disturbances naturally occuring from imperfections in the voltage clamp during VC runs as $\tau_{1/2}≲0.075$ s (see Fig CB in S1 Text), such that $\dot{V}\tau_{1/2}/\ln2\approx {\dot{V}}_{h}\tau_{1/2}/\ln2≲0.2$ mV. Thus, the systematic bias between $I_{vc,sm}(V_{h})$ in Fig 1B and the true steady state curve $I_{eq}(V)$ caused by dynamically changing $V_{h}$ is below measurement disturbances.

Deviation estimate (5) can also be tested *in silico*. Figs 3 and 4 emulate both VC and CC protocols with the Morris-Lecar model [33] (see Materials and methods, equation (10)), a biophysical model typically used for excitatory neurons of either class-I (Fig 3A) or class-II (Fig 4) excitability, and with the Wang-Buzsáki model (Fig 3B), which is a classical model of inhibitory interneuron [34] (see Materials and methods, equation (12)). Both models are of general form (1) with *j* = *n*_*j*_ = 1. For the chosen parameter set (see S1 Text), the curves $\left(I_{vc}(t),V_{h}(t)\right)$ and $\left(I_{eq}(V_{h}(t)),V_{h}(t)\right)$ are order *ε* (≈1%) apart.

Consequently, time profile $\left(I_{vc}(t),V_{h}(t)\right)$ follows closely the steady-state *I*–*V* curve (4) of *stable* steady states of the fast subsystem (1) with VC protocol $I_{ext}=g_{c}(V_{h}-V)$, treating $V_{h}$ as a parameter. This implies that faster ramp speeds are permissible when optimising trade-off between drift of cell properties and bias due to nonzero ramp speed. At high voltages the factor $I_{eq}^{\prime }(V)$ becomes large, such that estimate (5) predicts larger deviations for large $V_{h}$, as confirmed in Figs 1B, 2, AB, AC and AD in S1 Text.

We now connect the steady-state *I*–*V* curve (4) of the VC protocol to a curve of fast-subsystem equilibria of the CC protocol, which is in part unstable. To this end we recast the VC protocol in the form of a CC protocol with non-constant current ramp speed $\varepsilon \Delta_{I,vc}(t)$ and disturbances: the smoothed time profile $I_{vc,sm}(V_{h}(t))$ in Fig 1B of the VC run (thick, in blue/brown/red) equals the raw-data measured time profile $I_{vc}(t)$ (thin blue curve with fluctuations) plus disturbances $\eta_{vc}(t)$, defined by $\eta_{vc}(t)=I_{vc,sm}(V_{h}(t))\;-\;I_{vc}(t)$. After smoothing, the derivative $I_{vc,sm}^{\prime }(V_{h})$ w.r.t. $V_{h}$ is moderate (≲20 pA/mV in modulus at its maximum near $I_{db}$), such that during the VC protocol the external current $I_{ext}$ satisfies

$$I_{ext}\approx I_{vc,sm}+\eta_{vc},\;{\dot{I}}_{vc,sm}=\varepsilon \Delta_{I,vc}(t),$$ (6)

where $\Delta_{I,vc}(t)=I_{vc,sm}^{\prime }(V_{h}(t))\Delta_{V}$. Hence, $\Delta_{I,vc}(t)\approx I_{eq}^{\prime }(V_{h}(t))\Delta_{V}$, with upper bound $\max_{t}|\Delta_{I,vc}(t)|≲3.7$ pA/ms in the range of Fig 1B. Thus, $I_{vc,sm}$ is indeed still slow. So, except for disturbances $\eta_{vc}(t)$, the external current $I_{ext}$ is slowly varying according to a CC protocol with slowly time-varying speed $\varepsilon \Delta_{I,vc}(t)$, such that the VC protocol (2) is equivalent to the CC protocol (6) with disturbances $\eta_{vc}$.

This means that general model (1) with current $I_{ext}$ given in (6) with zero disturbances ($\eta_{vc}=0$) is a model for a CC protocol with driving current $I_{vc,sm}$, in contrast to the model with open-loop CC protocol (1), (3). Both models have the same fast subsystem (1) when setting ε=0 (i.e., for constant input current) and identifying $I_{h}$ and $I_{vc,sm}$. The respective fast-subsystem steady states $(V,x_{j}{)}_{j=1,\ldots ,N}$ satisfy

$$\sum_{j}I_{j}(x_{j},V)=I_{h}(=I_{vc,sm}),\;x_{j}=x_{\infty ,j}(V).$$ (7)

However, the two models differ by the nature of their respective slow variables $I_{h}$ and $I_{vc,sm}$: $I_{h}$ is an externally applied hold current for the open-loop CC protocol (3), while $I_{vc,sm}$ is a measured (and smoothed) current from the closed-loop feedback control $g_{c}(V_{h}\;-\;V)$ of the voltage source for (6). Thus, while the S-shaped steady-state curve $\left(I_{eq}(V),V\right)$ is identical for both models, it contains large unstable segments as a steady-state curve of open-loop CC protocol (1), (3), while it is always stable as a steady-state curve of closed-loop VC protocol (1), (6). The change in stability is caused by the disturbances $\eta_{vc}$, which are current adjustments generated by the feedback term in VC protocol (2), $I_{vc}=g_{c}(V_{h}\;-\;V)$. Along most of the curve $\left(I_{vc,sm}(V_{h}),V_{h}\right)$ the $\eta_{vc}$ are small fluctuations such that $I_{vc,sm}(V_{h})\approx I_{vc}(V_{h})$ and the feedback is approximately non-invasive [7]. Estimate (5) ensures that the measurements $I_{vc}(V)$ stay close to $I_{eq}(V)$. Therefore, we can conclude that the VC protocol (2) with slowly varying feedback reference signal $V_{h}$ reveals the entire family of steady states of a neuron (class I or II) with constant external current $I_{ext}$, both stable (observable) and unstable (non-observable, hidden). Consequently, the N-shaped *I*-*V* relations for class-I neurons reported in [19,20,23] equal S-shaped steady-state bifurcation diagrams for these neurons with respect to $I_{ext}$. They are tractable with a VC protocol where the current $I_{ext}$ is a sufficiently slowly varying feedback current with sufficiently small fluctuations $\eta_{vc}$. In particular, this allows us to detect and pass through fold bifurcations directly in the experiment.

**Fig 5:**
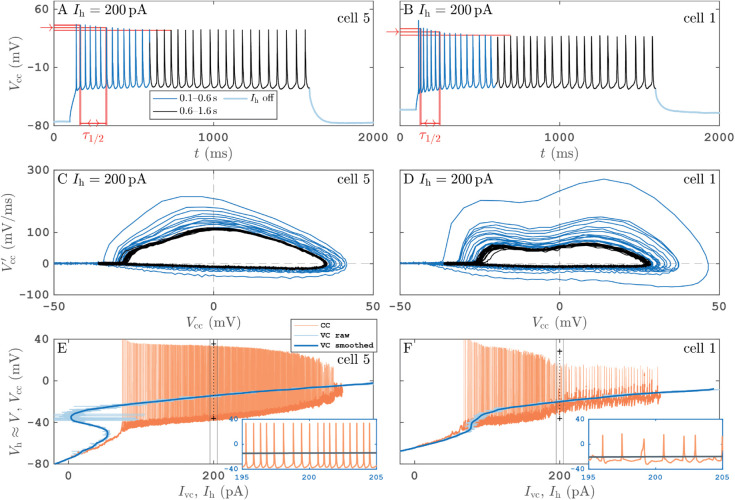
Periodic spiking responses to step-current protocols for PY cells 5 (left column) and 1 (right column). Panels A, B: time profiles of voltage responses $V_{cc}(t)$ from a current-clamp stimulation with a step to constant hold current $I_{h}=200$ pA from 0.1 to 1.6 s (0.1–0.6 s in blue, 0.6–1.6 s in black). Red markings show how half-decay time $\tau_{1/2}$ is extracted from voltage maxima during transients. Panels C, D: $\left(V_{cc},V_{cc}^{\prime }\right)$ phase-plane projection the time series from panels A, B, using a one-step finite-difference approximation of $V_{cc}^{\prime }(t)$ with color code matching panels A, B to distinguish transients and steady-state spiking. Panels E, F: reproduction of Figs 1A and AA in S1 Text without bifurcation or stability markings, respectively. The value $I_{h}=200$ pA (vertical dotted black line) and a 10 ms window around it (vertical solid black lines) are highlighted. The insets show a zoom into this 10 ms window around $I_{h}=200$ pA of panels E, F. Black crosses in panels E, F are minimum and maximum of steady-state spiking from panels A, B for comparison.

In contrast, during the CC open-loop protocol (3), when applying a slowly varying electrical current, the neuron dynamically transitions between its observable rest states and its observable (dynamically stable) spiking states (see Fig 1B orange time profile). The speed of variation $\varepsilon \Delta_{I}≲0.75\;\times 1\;0^{-3}$ pA/ms is such that $I_{ext}$ varies by 1 pA or less per spiking period. For a transient decay analysis we stimulate the neuron with larger current steps during a calibration phase before executing CC protocol runs. Fig 5A and 5B show the response to such a current step to $I_{ext}=200$ pA for cells 5 and 1. We observe that the transients in the step current response such as in Fig 5A and 5B in the stable spiking region have a half-time for decay toward the stable spikes

$$\tau_{1/2}≲0.2\text{ s.}$$ (8)

This half-time $\tau_{1/2}$ enters estimates for the bias caused by varying the hold current $I_{h}$ dynamically with speed $\varepsilon \Delta_{I}$. For example, the estimate for the bias in the minimum voltage $V_{\min}$ of the spike equals to first order in *ε*

$$\text{bias}(V_{\min})\approx V_{\min}^{\prime }(I_{h})\times \frac{d}{dt}I_{h}(t)\times \frac{\tau_{1/2}}{\ln2}.$$ (9)

We can see in Fig 5E and 5F that $V_{\min}(I_{h})$ changes in the spiking region with about 8 mV per 120 pA, so $V_{min}^{\prime }(I)\approx 0.07$ mV/pA. The current changes with dI/dt≈7.5 pA/s. Thus, to first order in *ε*, the error then amounts to:


$$\text{bias}(V_{\min})\approx V_{\min}^{\prime }(I_{h})\times \frac{d}{dt}I_{h}(t)\times \frac{\tau_{1/2}}{\ln2}\approx 0.14\text{ mV.}$$

Hence, the effect from changing the current $I_{h}$ dynamically is small (below visibility in bifurcation diagrams such as Fig 1B or Fig 5E and 5F. The linear bias estimate grows to infinity when the amplitude $V_{\max}(I_{h})$ envelope assumes a square-root like shape and $\tau_{1/2}$ goes to infinity, as is the case at Hopf bifurcations.

Thus, combining the VC protocol (6) for varying $I_{vc,sm}$, and the CC protocol (3) for varying $I_{h}$, enables us to interpret the data sets from both protocols in Fig 1B as a bifurcation diagram including unstable states.

#### Drift and intermittent dropouts.

In Fig 5E and 5F the black markers (+) at $I_{h}=200$ pA mark the maximum and minimum values of the voltage response (after transient) to the step-current stimulation, as shown in panels A and B. They indicate that for PY cell 5 there is quantitative agreement between the response to the CC protocol with slowly-varying applied current $I_{h}(t)$ and the response to the current step. There is less agreement for PY cell 1, between step response and the response to the CC protocol with slow variation of $I_{h}$. The time profile suggests that there are either different possible spiking responses and, hence, more bifurcations in the dynamic protocol, such as period-doubling bifurcations, or the condition of the PY cell has drifted between step response calibration and CC ramp.

Furthermore, the distances between $\left(I_{vc,sm}(t),V_{h}(t)\right)$ in the VC run and $\left(I_{h}(t),V_{cc}(t)\right)$ in the CC run for the same cell are visibly larger than predicted by eq (5) along parts of the curve corresponding to dynamically stable stationary states. This is due to the natural *drift* of the neuron’s physiological properties as it changes conductance properties (e.g., temperature, degradation,...). In S1 Text we show that over time.

Finally, for the experimental curves presented in Figs 1B, 1C, 2 and A in S1 Text, the disturbances $\eta_{vc}$ are not small in some unstable parts of the reported steady-state curve (e.g., near $V_{h}=-30$ mV in Fig 1B), caused by imperfect voltage clamping across the membrane. Fig CB in S1 Text shows a zoom demonstrating that the observed current spikes are indeed intermittent dropout events. Detailed simulations using Morris-Lecar model (10), shown in S1 Text Sect 3, reproduce these dropouts closely, if adds short current spikes and small-amplitude white noise to (10).

## Discussion

Tracking non-observable (hidden) states and their stability boundaries in experimental settings bridges the gap between real-world phenomena and nonlinear science. Specifically, closed-loop control methods with slow variations of feedback reference signals enable to dissect the underlying states of multi-scale complex systems. Following this strategy, we demonstrate the feasibility of tracking a family of neuronal steady states (stable and unstable) via variations of reference signal and reparameterizing the obtained curve by feedback current. The method is versatile in that it applies to different cell types, both pyramidal (PY) neurons (like in Fig 1), of excitability class I or II (Figs 3A and 4), and inhibitory interneurons (Fig 3B).

Our analysis and the resulting estimate for the nonzero-speed induced bias during the VC protocol in (5) indicates that for the steady states detected in VC ramps our ramp speed (less than 3 mV/s) does not create a bias that is notable when compared to drift of cell properties over time or fluctuations caused by external effects (such as the dropouts visible in Fig 1B). For the CC protocol and parameter areas were periodic spiking is stable, transient analysis for step current responses results in estimates such as (9), where bias caused by nonzero current ramp speed (≈7.5 pA/s) is also negligible. This implies that faster ramp speeds are permissible when optimising trade-off between drift of cell properties and bias due to nonzero ramp speed. The single-ramp nature of the CC protocol leaves the question of stability open for some parts of the steady-state branch. These results can be improved by varying the hold current $I_{h}$ non-monotonically and be switching from VC to CC protocol at selected parts of the steady-state branch determined by the VC protocol.

These results can be also extended to more complex neuronal states (e.g. limit cycles) by combining two recent advances, namely: 1. *dynamic-clamp* electrophysiology [2,36–40], which allows for a two-way real-time communication between a neuronal tissue (e.g. neuron, network or neuronal sub-processes) and a computer simulation of a component of the neuronal tissue. This introduces the full range of feedback control theory and engineering techniques into electrophysiology; 2. *Control-based continuation for experiments (CBCE*, which combines feedback control theory and pseudo-arclength numerical continuation for tracking solution branches of nonlinear systems directly from noisy experimental data [7]. Indeed, CBCE method has been successfully applied in various experimental systems, for instance, in mechanical vibration and buckling experiments [8], pedestrian flow experiments [9], cylindrical pipe flow simulations [11] and feasibility studies for synthetic gene networks [12,13]. Noteworthy, the methodology presented in this work can be seen as a special case of CBCE. Future work will focus on employing the full CBCE to track more complex neuronal states, for instance, unstable spiking states in single neurons or unstable traveling-wave states in neural tissue using micro-electrode arrays (MEA). This is crucial as it will allow us to validate computational models by comparing their numerical bifurcation diagrams with experimental ones; it will also help obtaining better model fitting based on both voltage-clamp and current-clamp data. This approach will have direct impact in experimental labs enabling biologists to have access to novel states with which to carry out new experimental paradigms. We envisage this extended protocol could be used to develop next-generation closed-loop deep-brain stimulation devices to treat certain brain diseases such as epilepsy [41], where it could help monitor control the excitability threshold of key neural populations in real time.

## Materials and methods

### Ethics statement

All animal treatments were authorized by the Sechenov Institute of Evolutionary Physiology and Biochemistry Bioethics Committee (protocol no. 1-7/2022, 27 January 2022) and adhered to the European Community Council Directive 86/609/EEC.

### Protocols

Male Wistar rats were used in this study (age P21, N=8 animals). For the bifurcation diagrams (see Figs 1B and A in S1 Text), 4 of these 8 rats were used. PY Cells 1-3 were recorded from different animals, PY cells 4-5 were recorded from the same animal. To study the effects of hysteresis (see Fig 2) 20 neurons were recorded from the 4 remaining animals (221222, 230110, 230111, 230112). The control group were 6 neurons from 2 rats (230110, 230112). The QX group were 8 neurons from 4 rats (221222, 230110, 230111, 230112). The QX+Cd group were 6 neurons from 4 rats (221222, 230110, 230111, 230112).

The use and handling of animals was performed in accordance with the European Community Council Directive 86/609/EEC. Horizontal 300-μm-thick brain slices were prepared as described in our previous studies [42]. The slices contained the hippocampus and the adjacent cortical regions and were kept in the artificial cerebrospinal fluid (ACSF) with the following composition (in mM): 126 NaCl, 24 NaHCO_3_, 2.5 KCl, 2 CaCl_2_, 1.25 NaH_2_PO_4_, 1 MgSO_4_, 10 glucose (bubbled with 95% O2/5% CO_2_ gas mixture). All the listed chemicals were purchased from Sigma-Aldrich (St. Louis, MO, USA).

We performed the whole-cell patch-clamp recordings of the principal neurons in the entorhinal cortex. Neurons within the slices were visualized using a Zeiss Axioscop 2 microscope (Zeiss, Oberkochen, Germany), equipped with a digital camera (Grasshopper 3 GS3-U3-23S6M-C; FLIR Integrated Imaging Solutions Inc., Wilsonville, OR, USA) and differential interference contrast optics.

Patch pipettes were produced from borosilicate glass capillaries (Sutter Instrument, Novato, CA, USA) and filled with one of the following pipette solutions. A potassium gluconate-based pipette solution had the following composition (in mM): 136-K-Gluconate, 10 NaCl, 10 HEPES, 5 EGTA, 4 ATP-Mg, 0.3 GTP. For control experiments described in Section SI4, the intracellular block of the voltage-gated sodium channels was required, for which we used the solution with added QX314 (Alamone labs, Jerusalem, Israel), which had the following composition (in mM): 130-K-Gluconate, 10 HEPES, 6 QX314, 6 KCl, 5 EGTA, 4 ATP-Mg, 2 NaCl, 0.3 GTP. The pH of both solutions was adjusted to 7.25 with KOH. The resistance of filled patch-pipettes was 3-4 MΩ.

A Multiclamp 700B (Molecular Devices, Sunnyvale, CA, USA) patch-clamp amplifier, a NI USB-6343 A/D converter (National Instruments, Austin, TX, USA) and WinWCP 5 software (SIPBS, UK) were used to obtain the electrophysiological data. The recordings were lowpass filtered at 10 kHz and sampled at 20-30 kHz. The access resistance was less than 15 MΩ and remained stable during the recordings. The liquid junction potential was not compensated for. The flow rate of ACSF in the recording chamber was 5 ml/min. The recordings were performed at 30°C.

Specifically for the voltage-clamp protocol. We note the CV-7B headstage has four different feedback resistors (Rf): 50 MΩ, 500 MΩ, 5 GΩ, and 50 GΩ. The Rf determines the maximum currents that can be recorded or injected. In voltage-clamp mode it is generally recommended to use the largest possible value of Rf (larger Rf results in less noise), though high values can result in current saturation. Since in our preparation the electrical currents varied between 50-2000 pA (several nA for the potassium ion currents at positive holding potentials), we chose Rf = 500 MΩ (i.e. feedback gain *g*_*c*_ = 2 nS).

We applied first VC and then CC protocol to 5 neurons in the entorhinal cortex of 4 male Wistar rats [42]. We first performed the VC neuronal recordings varying the hold voltage $V_{h}$ from –80 mV to  + 30 mV slowly with ${\dot{V}}_{h}=1.83$ mV/s, while measuring current, called $I_{vc}$ in Figs 1B and 5C. Subsequently, for the CC recordings we first determine the minimal injected current required to induce a depolarization block of action potential generation ($I_{db}$ in Fig 1B, upper limit of current input where firing occurs). Then we injected hold current $I_{h}$, gradually increasing from 0 pA to $I_{db}$ during 60 s, such that ${\dot{I}}_{h}$ is in the range 6.6…7.4 pA/s for the 5 neurons, while recording voltage $V_{cc}(t)$. See S1 Text for further checks (e.g., for hysteresis).

### Computational models

We applied the VC and the CC protocols, with slow variations in the feedback reference signal and in the applied current, respectively, to the classical neuron model adapted to many contexts, namely the Morris-Lecar model [33] (ML), and to a standard neuron model that was specifically designed to account for inhibitory neural activity, namely the Wang-Buzsáki model [34] (WB). Parameter values are given in Tables 1 and 2 below, respectively.

**Table 1:** Parameter values for the Morris-Lecar model (10).

parameter	*C*	$g_{L}$	$V_{L}$	$g_{Ca}$	$V_{Ca}$	$g_{K}$	$V_{K}$	$g_{c}$	ϕ	$V_{1}$	$V_{2}$	$V_{3}$	$V_{4}$
unit	pF	nS	mV	nS	mV	nS	mV	nS		mV	mV	mV	mV
value class-I	20	2	-60	4.0	120	12	-84	40	0.067	-1.2	18	12	17.4
value class-II	20	2	-60	4.4	120	12	-84	150	0.040	-1.2	18	2	30.0

**Table 2:** Parameter values for the Wang-Buzsáki model (12).

parameter	*C*	$g_{L}$	$V_{L}$	$g_{Na}$	$V_{Na}$	$g_{K}$	$V_{K}$	$g_{c}$	ϕ
unit	pF	nS	mV	nS	mV	nS	mV	nS	
value	1	0.1	–65	35.0	55	9	–90	20	5

The ML equations are as follows

$$C\dot{V}=-g_{L}(V-V_{L})-g_{Ca}m_{\infty }(V)(V-V_{Ca})-g_{K}w(V-V_{K})+g_{c}(V_{h}-V),\;\dot{w}=\phi \frac{w_{\infty }(V)-w}{\tau_{w}(V)},$$ (10)

with the following voltage-dependent (in)activation and time-constant functions:

$$m_{\infty }(V)=0.5(1+\tanh((V-V_{1})/V_{2})),\;w_{\infty }(V)=0.5(1+\tanh((V-V_{3})/V_{4})),\;\tau_{w}(V)=\cosh((V-V_{3})/(2V_{4}){)}^{-1}.$$ (11)

To obtain Figs 3A and 4, we have used the following parameter values.

Finally, for both the VC protocol with slow variation, and the CC protocol with slow variation, the speed of the variation was chosen to be equal to ε=0.01. Note that *g*_*c*_ can be decreased to 7, which is in the same order of magnitude as the experimental one. Moreover, the capacitance chosen for the model simulations is on the same order of magnitude as observed in the experiments. Nevertheless, the key point to note is that we are not aiming for quantitative agreement since this is a conductance-based phenomenological model.

The WB equations are as follows

$$C\dot{V}=-g_{L}(V-V_{L})-g_{Na}m_{\infty }^{3}(V)h(V-V_{Na})-g_{K}n^{4}(V-V_{K})+g_{c}(V_{h}-V),\;\dot{h}=\phi \frac{h_{\infty }(V)-h}{\tau_{h}(V)},\;\dot{n}=\phi \frac{n_{\infty }(V)-n}{\tau_{n}(V)},$$ (12)

with the following voltage-dependent (in)activation and time-constant functions:

$$\alpha_{m}(V)=0.1(V+35)/(1-\exp(-0.1(V+35)))\;\beta_{m}(V)=4.0\exp(-0.0556(V+60))\;\alpha_{h}(V)=0.07\exp(-0.05(p+58))\;\beta_{h}(V)=1/(1+\exp(-0.1(V+28)))\;\alpha_{n}(V)=0.01(V+34)/(1-\exp(-0.1(V+34)))\;\beta_{n}(V)=0.125\exp(-0.0125(V+44))\;p_{\infty }(V)=\alpha_{p}(V)/(\alpha_{p}(V)+\beta_{p}(V)),\;p\in \{m,h,n\},\;\tau_{p}(V)=1/(\alpha_{p}(V)+\beta_{p}(V)),\;p\in \{m,h,n\}$$ (13)

To obtain Fig 3B, we have used the following parameter values.

Finally, for both the VC protocol with slow variation, and the CC protocol with slow variation, the speed of the variation was chosen to be equal to ε=0.01.

## References

[1] Ori H, Marder E, Marom S. Cellular function given parametric variation in the Hodgkin and Huxley model of excitability. Proc Natl Acad Sci U S A. 2018;115(35):E8211–8. doi: 10.1073/pnas.1808552115 30111538 PMC6126753

[2] Ori H, Hazan H, Marder E, Marom S. Dynamic clamp constructed phase diagram for the Hodgkin and Huxley model of excitability. Proc Natl Acad Sci U S A. 2020;117(7):3575–82. doi: 10.1073/pnas.1916514117 32024761 PMC7035484

[3] Levenstein D, Buzsáki G, Rinzel J. NREM sleep in the rodent neocortex and hippocampus reflects excitable dynamics. Nat Commun. 2019;10(1):2478. doi: 10.1038/s41467-019-10327-5 31171779 PMC6554409

[4] Hesse J, Schleimer J-H, Maier N, Schmitz D, Schreiber S. Temperature elevations can induce switches to homoclinic action potentials that alter neural encoding and synchronization. Nat Commun. 2022;13(1):3934. doi: 10.1038/s41467-022-31195-6 35803913 PMC9270341

[5] Sip V, Hashemi M, Dickscheid T, Amunts K, Petkoski S, Jirsa V. Characterization of regional differences in resting-state fMRI with a data-driven network model of brain dynamics. Sci Adv. 2023;9(11):eabq7547. doi: 10.1126/sciadv.abq7547 36930710 PMC10022900

[6] Ladenbauer J, McKenzie S, English DF, Hagens O, Ostojic S. Inferring and validating mechanistic models of neural microcircuits based on spike-train data. Nat Commun. 2019;10(1):4933. doi: 10.1038/s41467-019-12572-0 31666513 PMC6821748

[7] Sieber J, Gonzalez-Buelga A, Neild SA, Wagg DJ, Krauskopf B. Experimental continuation of periodic orbits through a fold. Phys Rev Lett. 2008;100(24):244101. doi: 10.1103/PhysRevLett.100.244101 18643585

[8] Renson L, Shaw AD, Barton DAW, Neild SA. Application of control-based continuation to a nonlinear structure with harmonically coupled modes. Mechanical Systems and Signal Processing. 2019;120:449–64. doi: 10.1016/j.ymssp.2018.10.008

[9] Panagiotopoulos I, Starke J, Just W. Control of collective human behavior: Social dynamics beyond modeling. Phys Rev Res. 2022;4(4):043190.

[10] Böttcher L, Wallner H, Kruse N, Just W, Barke I, Starke J, et al. Exposing hidden periodic orbits in scanning force microscopy. Commun Phys. 2025;8(1):1–9. doi: 10.1038/s42005-025-01958-w

[11] Willis AP, Duguet Y, Omelchenko O, Wolfrum M. Surfing the edge: using feedback control to find nonlinear solutions. J Fluid Mech. 2017;831:579–91. doi: 10.1017/jfm.2017.656

[12] de Cesare I, Salzano D, di Bernardo M, Renson L, Marucci L. Control-based continuation: a new approach to prototype synthetic gene networks. ACS Synth Biol. 2022;11(7):2300–13. doi: 10.1021/acssynbio.1c00632 35729740 PMC9295158

[13] Blyth M, Tsaneva-Atanasova K, Marucci L, Renson L. Numerical methods for control-based continuation of relaxation oscillations. Nonlinear Dyn. 2023;111(9):7975–92. doi: 10.1007/s11071-023-08288-y

[14] Phillips CL, Harbor RD. Feedback control systems. 4th ed. Prentice-Hall, Inc.; 1999.

[15] Allgower EL, Georg K. Introduction to numerical continuation methods. SIAM; 2003.

[16] Cole KS. Ions, potentials and the nerve impulse. In: Shedlovsky T, editor. Electrochemistry in biology and medicine; 1955. p. 121–40.

[17] Hodgkin AL, Huxley AF. Currents carried by sodium and potassium ions through the membrane of the giant axon of Loligo. J Physiol. 1952;116(4):449–72. doi: 10.1113/jphysiol.1952.sp004717 14946713 PMC1392213

[18] Schaefer AT, Angelo K, Spors H, Margrie TW. Neuronal oscillations enhance stimulus discrimination by ensuring action potential precision. PLoS Biol. 2006;4(6):e163. doi: 10.1371/journal.pbio.0040163 16689623 PMC1459879

[19] Schwindt P, Crill W. Voltage clamp study of cat spinal motoneurons during strychnine-induced seizures. Brain Res. 1981;207(2):471–5. 7248751 10.1016/0006-8993(81)90669-7

[20] Fishman HM, Macey RI. The N-shaped current-potential characteristic in frog skin. 3. Ionic dependence. Biophys J. 1969;9(2):151–62. doi: 10.1016/s0006-3495(69)86376-9 5764225 PMC1367424

[21] Johnston D, Hablitz JJ, Wilson WA. Voltage clamp discloses slow inward current in hippocampal burst-firing neurones. Nature. 1980;286(5771):391–3. doi: 10.1038/286391a0 7402320

[22] Blatt MR. Potassium-dependent, bipolar gating of K channels in guard cells. J Membr Biol. 1988;102:235–46.

[23] Vervaeke K, Hu H, Graham LJ, Storm JF. Contrasting effects of the persistent Na+ current on neuronal excitability and spike timing. Neuron. 2006;49(2):257–70. doi: 10.1016/j.neuron.2005.12.022 16423699

[24] Del Negro CA, Hsiao CF, Chandler SH, Garfinkel A. Evidence for a novel bursting mechanism in rodent trigeminal neurons. Biophys J. 1998;75(1):174–82. doi: 10.1016/S0006-3495(98)77504-6 9649377 PMC1299689

[25] Qian K, Yu N, Tucker KR, Levitan ES, Canavier CC. Mathematical analysis of depolarization block mediated by slow inactivation of fast sodium channels in midbrain dopamine neurons. J Neurophysiol. 2014;112(11):2779–90. doi: 10.1152/jn.00578.2014 25185810 PMC4254877

[26] Rinzel J. A formal classification of bursting mechanisms in excitable systems. In: Mathematical topics in population biology, morphogenesis and neurosciences (Proceedings of an International Symposium held in Kyoto, November 10-15, 1985). vol. 71 of Lect. Notes Biomath. Springer; 1987. p. 267–81.

[27] Baer SM, Erneux T. Singular Hopf bifurcation to relaxation oscillations. SIAM J Appl Math. 1986;46(5):721–39. doi: 10.1137/0146047

[28] Lindner B. Effects of noise in excitable systems. Physics Reports. 2004;392(6):321–424. doi: 10.1016/j.physrep.2003.10.015

[29] Dovzhenok A, Kuznetsov AS. Exploring neuronal bistability at the depolarization block. PLoS One. 2012;7(8):e42811. doi: 10.1371/journal.pone.0042811 22900051 PMC3416767

[30] Rush ME, Rinzel J. Analysis of bursting in a thalamic neuron model. Biol Cybern. 1994;71(4):281–91. doi: 10.1007/BF00239616 7948220

[31] Magrans de Abril I, Yoshimoto J, Doya K. Connectivity inference from neural recording data: challenges, mathematical bases and research directions. Neural Netw. 2018;102:120–37. doi: 10.1016/j.neunet.2018.02.016 29571122

[32] Izhikevich EM. Dynamical systems in neuroscience. MIT Press; 2007.

[33] Morris C, Lecar H. Voltage oscillations in the barnacle giant muscle fiber. Biophys J. 1981;35(1):193–213. doi: 10.1016/S0006-3495(81)84782-0 7260316 PMC1327511

[34] Wang XJ, Buzsáki G. Gamma oscillation by synaptic inhibition in a hippocampal interneuronal network model. J Neurosci. 1996;16(20):6402–13. doi: 10.1523/JNEUROSCI.16-20-06402.1996 8815919 PMC6578902

[35] Fenichel N. Geometric singular perturbation theory for ordinary differential equations. J Differ Eq. 1979;31(1):53–98.

[36] Sharp AA, O’Neil MB, Abbott LF, Marder E. Dynamic clamp: computer-generated conductances in real neurons. J Neurophysiol. 1993;69(3):992–5. doi: 10.1152/jn.1993.69.3.992 8463821

[37] Marder E, Abbott LF, Turrigiano GG, Liu Z, Golowasch J. Memory from the dynamics of intrinsic membrane currents. Proc Natl Acad Sci U S A. 1996;93(24):13481–6. doi: 10.1073/pnas.93.24.13481 8942960 PMC33634

[38] Chizhov AV, Malinina E, Druzin M, Graham LJ, Johansson S. Firing clamp: a novel method for single-trial estimation of excitatory and inhibitory synaptic neuronal conductances. Front Cell Neurosci. 2014;8:86. doi: 10.3389/fncel.2014.00086 24734000 PMC3973923

[39] Kirst C, Ammer J, Felmy F, Herz A, Stemmler M. Fundamental structure and modulation of neuronal excitability: synaptic control of coding, resonance, and network synchronization. BioRxiv. 2015; 022475.

[40] Pfeiffer P, Barreda Tomás FJ, Wu J, Schleimer J-H, Vida I, Schreiber S. A dynamic clamp protocol to artificially modify cell capacitance. Elife. 2022;11:e75517. doi: 10.7554/eLife.75517 35362411 PMC9135398

[41] Breakspear M, Roberts JA, Terry JR, Rodrigues S, Mahant N, Robinson PA. A unifying explanation of primary generalized seizures through nonlinear brain modeling and bifurcation analysis. Cereb Cortex. 2006;16(9):1296–313. doi: 10.1093/cercor/bhj072 16280462

[42] Amakhin DV, Soboleva EB, Chizhov AV, Zaitsev AV. Insertion of calcium-permeable AMPA receptors during epileptiform activity in vitro modulates excitability of principal neurons in the rat entorhinal cortex. Int J Mol Sci. 2021;22(22):12174. doi: 10.3390/ijms222212174 34830051 PMC8621524

